# ACE2 Expression in the Cat and the Tiger Gastrointestinal Tracts

**DOI:** 10.3389/fvets.2020.00514

**Published:** 2020-08-13

**Authors:** Roberto Chiocchetti, Giorgia Galiazzo, Federico Fracassi, Fiorella Giancola, Marco Pietra

**Affiliations:** Department of Veterinary Medical Sciences (UNI EN ISO 9001:2008), Alma Mater Studiorum University of Bologna, Bologna, Italy

**Keywords:** angiotensin-converting enzyme 2, COVID-19, feline, immunohistochemistry, SARS-CoV-2

## Abstract

Angiotensin-converting enzyme 2 (ACE2) has been identified as the functional receptor for Severe Acute Respiratory Syndrome—Coronavirus−2 (SARS-CoV-2). It has been identified in the human gastrointestinal tract (GIT), and SARS-CoV-2 has been isolated in human and animal fecal samples. The aim of the present study was to investigate the expression of ACE2 in the gastrointestinal tract of domestic (cat) and wild (tiger) felines. Samples of the pylorus, duodenum, and distal colon were collected from six cats and one tiger. The tissues were processed for immunofluorescence assay with an anti-human ACE2 antibody. Angiotensin-converting enzyme 2 was widely expressed in the gastrointestinal mucosa of the cats and the tiger. In both the species, ACE2-immunoreactivity (ACE2-IR) was expressed by the mucosal epithelial cells of the GIT and by the enteric neurons. In the cats, ACE2-IR was also expressed by the smooth muscle cells of the blood vessels and the *tunica muscularis*. The expression of the ACE2 receptor in enteric neurons may support the potential neurotropic properties of SARS-CoV-2. Although the evidence of ACE2-IR in the feline GIT does not necessarily indicate the possibility of viral replication and SARS-CoV-2 spread with stool, the findings in the present study could serve as an anatomical basis for additional studies considering the risk of the SARS-CoV-2 fecal-oral transmission between cats/felids, and between cats/felids and humans.

## Introduction

In the intestinal tract, angiotensin-converting enzyme 2 (ACE2) plays significant roles in amino acid homeostasis, innate immune responses, and intestinal microbiota regulation (1).

In addition to its physiological roles, ACE2 has been identified as the functional receptor for Severe Acute Respiratory Syndrome—Coronavirus−2 (SARS-CoV-2), the RNA virus responsible for Covid-19 (2–4). The spike proteins of SARS-CoV-2 associate with the ACE2 of sensitive cells to mediate infection of their target cells, after which viral replication begins in the cytoplasm.

The lungs seem to be the main target of SARS-CoV-2 in which the virus causes severe respiratory syndrome (5). However, it has been shown that ACE2 is also expressed in the epithelial cells of the human gastrointestinal tract (GIT) (6, 7). In addition, viral nucleocapsid proteins have been found in the epithelial cells of the human stomach, duodenum, and rectum (5), and SARS-CoV-2 has been isolated from stool samples (8). To reinforce the possibility of a fecal-oral route of viral transmission, it has been reported that ~10% of Covid-19 patients suffer from gastrointestinal symptoms which often precede fever, dry cough, and dyspnea (8), and that SARS-CoV-2 RNA persists in the stool specimens of about 20% of patients after respiratory symptoms had improved (5).

Taken together, this evidence supports the hypothesis that the digestive system, rather than the respiratory system, may serve as an alternative route of infection between humans and, perhaps, between infected animals and humans.

The new coronavirus has, with high probability, a zoonotic origin (9) and although SARS-CoV-2 is thought to have originated in bats, its intermediate host before transmitting to humans is not clear (10, 11). Different species of mammals (and birds) may be potential intermediate hosts for SARS-CoV-2. There is evidence that SARS-CoV-2 could utilize the ACE2 of Chinese horseshoe bats, civets, and swine (4); other reports have predicted that SARS-CoV-2 could utilize the ACE2s of other mammals, such as ruminants, hamsters, pangolins, and also cats (10, 12).

Domestic cats, a species in close contact with humans, are more susceptible to SARS-CoV-2 infection in comparison with other species, such as dogs and pigs in which the virus replicates poorly (11). Although there are still few studies regarding SARS-CoV-2 infection in cats, there is evidence of SARS-CoV-2 transmission between cats (11) in which the virus has been found in oral, nasal, and fecal samples (13). Moreover, reports indicate the transmission of SARS-CoV-2 not only from humans to domestic cats but also from human to lions and tigers in zoos (11, 14). A naturally infected cat (with infection transmitted by its Covid-19-positive owner) showed severe gastrointestinal (diarrhea and vomiting) and respiratory (dyspnea) clinical signs (15, 16).

Taken together, this evidence indicates the urgency of additionally investigating the potential chain of human-cat and also human–cat–human transmission of SARS-CoV-2. Of all the strategies utilized in preventing the spread of the virus, the prevention of fecal-oral transmission should be taken into careful consideration.

Since the necessary condition for the enteric multiplication of SARS-CoV-2 is represented by the expression of its election receptor on the host cells, the present study immunohistochemically investigated the localization of ACE2 in the GIT of feline domestic (cat) and wild (tiger) species.

## Methods

### Animals

The stomachs, duodenums, and distal colons were collected from six half-breed cats and one tiger from a zoo which died spontaneously or were euthanized for humane reasons due to different diseases (Table 1), and their tissues were collected following owner permission. The cats did not show gross alterations of the intestinal wall. The tiger showed moderate-to-severe hyperemia, mainly in the duodenum.

**Table 1:** Animals included in the study.

**Animals**	**Age**	**Sex**	**Species**	**Breed**	**Cause of death**
# 1	3 years	M	Cat (*Felis catus*)	Half-breed	Head traumaanitha (car accident)
# 2	4 years	F	Cat (*Felis catus*)	Half-breed	Head traumaanitha (car accident)
# 3	12 years	M	Cat (*Felis catus*)	Half-breed	Lymphoma
# 4	12 years	M	Cat (*Felis catus*)	Half-breed	Iliac thrombosis
# 5	12 years	M	Cat (*Felis catus*)	Half-breed	Oral neoplasia
# 6	13 years	M	Cat (*Felis catus*)	Half-breed	Lymphoma
# 7	17 years	F	Tiger (*Panthera tigris*)	Sumatrae	Chronic proliferative gastritis

Since the antibody anti-ACE2 employed in the present study is human-specific, a human submucosal wholemount preparation of descending colon was used as positive control, previous donator consent. Wholemount preparation was analyzed using prevalidated immunohistochemical protocols (17).

### Tissue Collection

The stomachs, duodenums, and distal colons were harvested within 1 h of the animals' deaths and were longitudinally opened along the great curvature (stomach) and the mesenteric border (intestine). The tissues were then washed in phosphate-buffered saline (PBS), gently pinned on wood board (thickness 5 mm), and fixed in 2% paraformaldehyde containing 0.2% picric acid in 0.1 M sodium phosphate buffer (pH 7.0) at +4°C for 48 h. After rinsing in PBS, the tissues were stored in PBS containing 30% sucrose and 0.1% sodium azide (pH 7.4) at +4°C.

Pieces of tissues (2.0 × 0.5 cm) were subsequently cut, transferred to a mixture of PBS–sucrose–azide and OCT compound (Tissue Tek®, Sakura Finetek Europe, Alphen aan den Rijn, the Netherlands) at a ratio of 1:1 (overnight), and then embedded in 100% OCT. The tissues were frozen, mounted in Tissue Tek® mounting medium, and sectioned at 14–16 μm on a cryostat. The sections were collected on poly-L-lysine coated slides, which were later processed for immunohistochemistry.

### Immunofluorescence

The cryosections were hydrated in PBS and processed for immunostaining. In order to block non-specific bindings, the sections were incubated in a solution containing 20% normal donkey serum (Colorado Serum Co., Denver, CO, USA), 0.5% Triton X-100 (Sigma Aldrich, Milan, Italy, Europe), and bovine serum albumin (1%) in PBS for 1 h at room temperature (RT). For single immunostaining, the cryosections were incubated in a humid chamber overnight at RT with antibody rabbit anti-human ACE2 (1:100; orb582208, Biorbyt, Cambridge, UK). For double immunostaining, the cryosections were incubated with rabbit anti-ACE and mouse anti-HuC/HuD (1:200; A-21271, Thermo Fisher Scientific, Waltham, MA USA) antibodies. The antibody anti-HuC/HuD was utilized as a pan-neuronal marker in order to identify the enteric neurons. The primary antibodies were diluted in 1.8% NaCl in 0.01M PBS, containing 0.1% sodium azide. After washing the sections in PBS (3 × 10 min), they were incubated for 1 h at RT in a humid chamber with the secondary antibodies (Donkey anti-rabbit IgG Alexa 488, 1:1,000, A-21206, Thermo Fisher; Donkey anti-mouse IgG Alexa 594, 1:500, A-21203, Thermo Fisher) diluted in PBS. The cryosections were then washed in PBS (3 × 10 min) and mounted in buffered glycerol at pH 8.6 with 4′, 6-diamidino-2-phenylindole—DAPI- (Santa Cruz Biotechnology, CA, USA).

The preparations were examined by the same observer (Dr. R. Chiocchetti) using a Nikon Eclipse Ni microscope equipped with the appropriate filter cubes to differentiate the fluorochromes used. The qualitative analysis of ACE2-IR was performed on high power fields (40×, longitudinal sections). The images were recorded using a Nikon DS-Qi1Nc digital camera and NIS Elements software BR 4.20.01 (Nikon Instruments Europe BV, Amsterdam, Netherlands). Slight adjustments to contrast and brightness were made using Corel Photo Paint, and the figure panels were prepared using Corel Draw (Corel Photo Paint and Corel Draw, Ottawa, ON, Canada).

## Results

### ACE2 Immunoreactivity in the Cats

**Figure 1:**
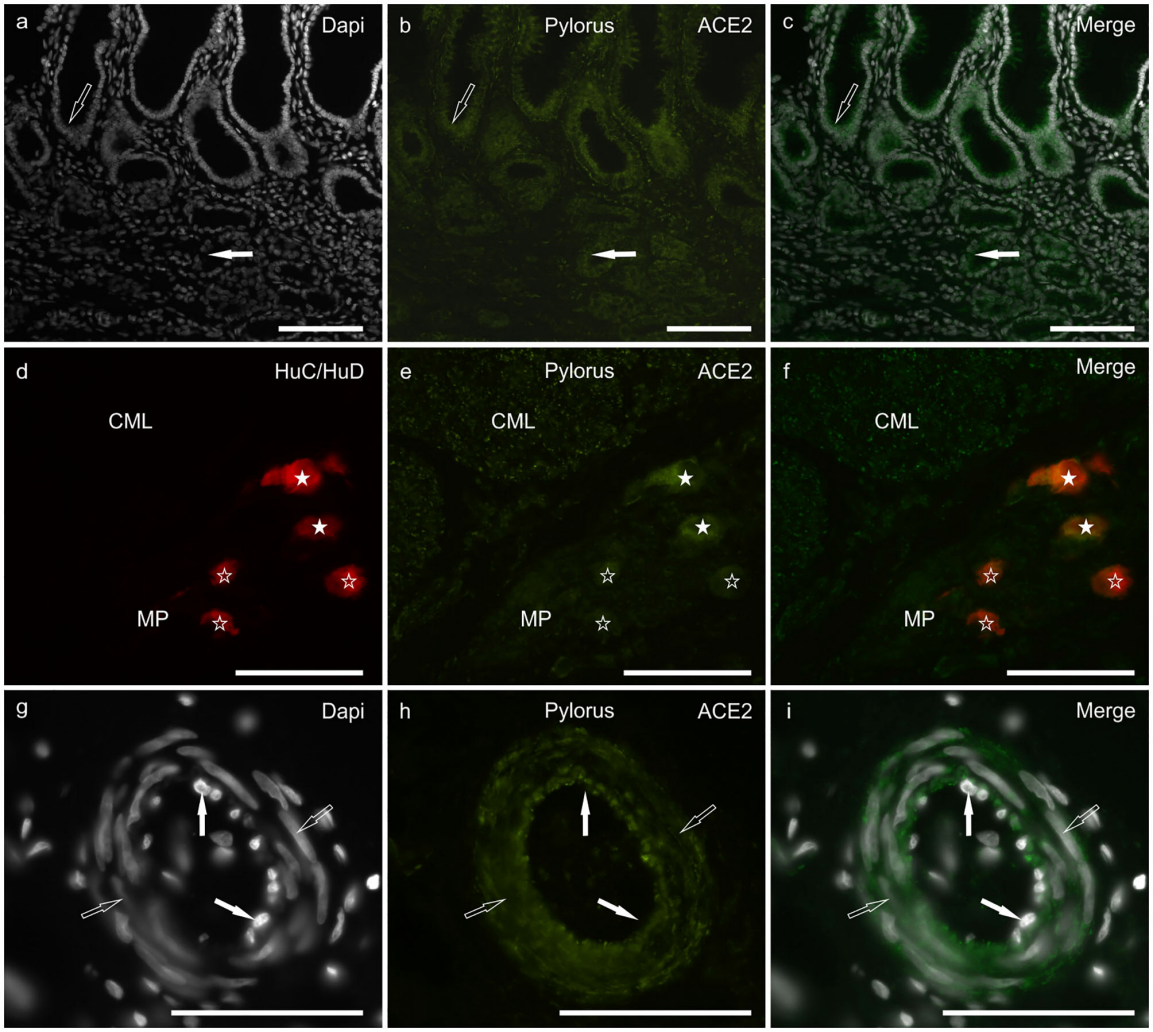
Photomicrographs of cryosections of a cat pylorus **(a–i)** showing angiotensin-converting enzyme 2 immunoreactivity (ACE2-IR) in the gastric mucosa **(a–c)**, myenteric plexus (MP) neurons **(d–f)** and blood vessels **(g–i)**. **(a–c)** Moderate ACE2-IR was expressed by the mucosal epithelial cells (open arrow) and secretory cells of the pyloric glands (white arrow). **(d–f)** White stars indicate two MP neurons expressing bright ACE2-IR which were also immunolabeled for the pan-neuronal marker HuC/HuD. Open stars indicate three MP neurons showing weak ACE2-IR. The smooth muscle cells of the *tunica muscularis* also showed moderate ACE2-IR as seen in the circular muscle layer (CML). **(g–i)** The smooth muscle cells (open arrows) of the blood vessels showed moderate ACE2-IR whereas ACE2-IR was undetectable in the endothelial cells (white arrows). Bar: 100 μm.

Moderate and granular ACE2 immunoreactivity (ACE2-IR) was expressed by the cytoplasm of the gastric mucosal epithelial cells and the secretory cells of the pyloric glands (Figures 1a–c). It was also displayed by the smooth muscle cells of the *muscularis mucosae* and *tunica muscularis* (Figures 1d–f), myenteric plexus (MP) neurons (Figures 1d–f), and the smooth muscle cells of the blood vessels (Figures 1g–i).

**Figure 2:**
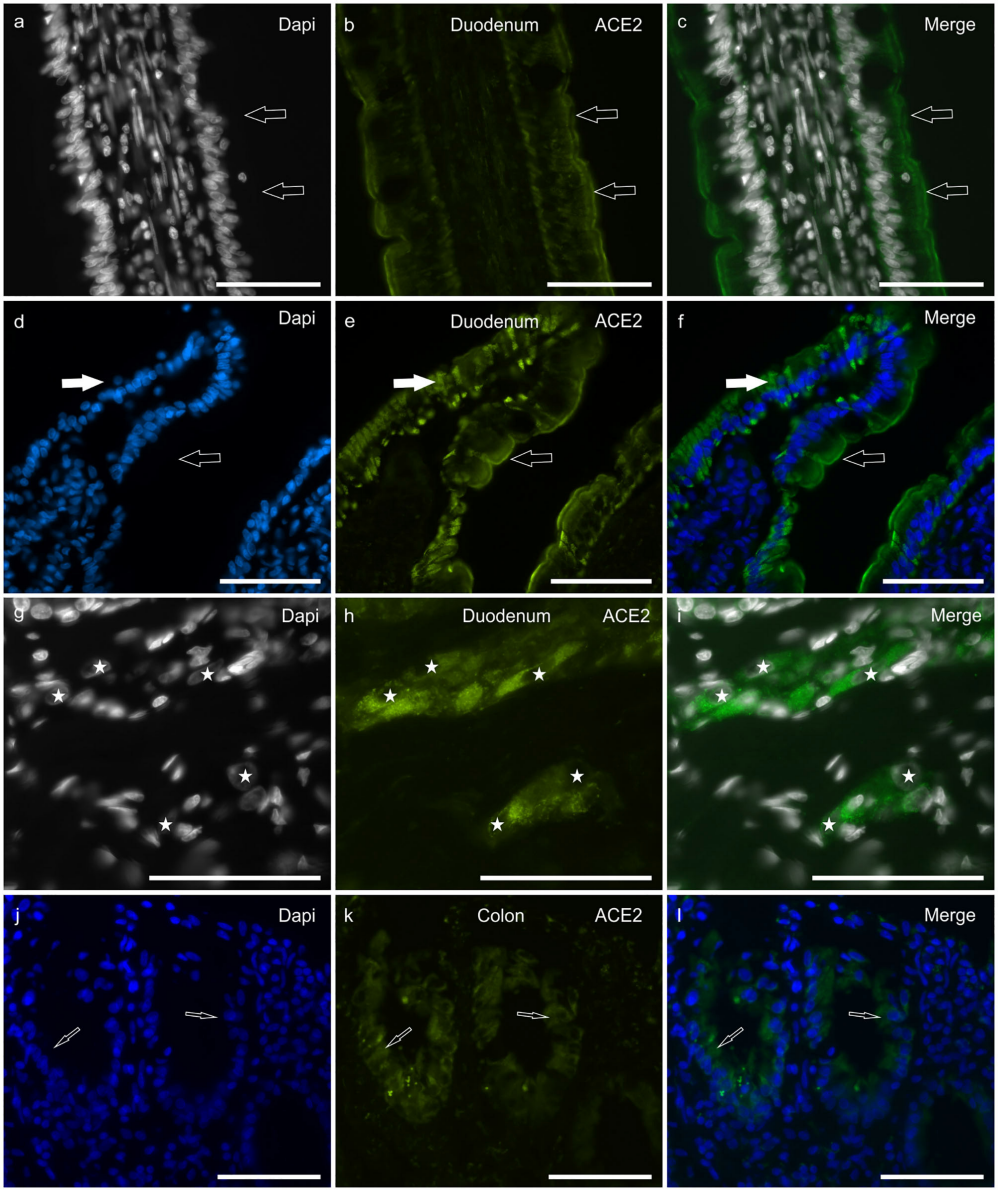
Photomicrographs of cryosections of a cat duodenum **(a–i)** and colon **(j–l)** showing angiotensin-converting enzyme 2 immunoreactivity (ACE2-IR) in the duodenal mucosa **(a–f)** and submucosal plexus (SMP) neurons **(g–i)**. **(a–c)** The arrows indicate ACE2-IR expressed by the brush border of enterocytes. **(d–f)** Open arrows indicate ACE2-IR expressed by the brush border of enterocytes; white arrows indicate the cytoplasmic distribution of the ACE2 immunolabeling. **(g–i)** Stars indicate the nuclei of SMP neurons showing bright and granular ACE2-IR. Bar: 100 μm.

In the duodenum, bright ACE2-IR was displayed by the brush border (Figures 2a–c) and the cytoplasm (Figures 2d–f) of the enterocytes, whereas it was undetectable or absent in goblet cells. The ACE2 immunolabeling was brighter in cells distributed along the villi than in the crypt cells. Moderate ACE2-IR was expressed by the secretory cells of the submucosal glands (Brunner's glands) (data not shown). The smooth muscle cells of the *muscularis mucosae* and *tunica muscularis* showed moderate ACE2-IR. The cytoplasm of intramural neurons distributed in the MP and submucosal plexus (SMP) showed moderate-to-bright ACE2-IR (Figures 2g–i).

In the colon, ACE2-IR was expressed by the epithelial cells of the crypts (Figures 2g–l) and by the other cell types described above for the duodenum; however, in the colon, the ACE2 immunolabeling was weaker when compared to that observed in the duodenum.

ACE2-IR was expressed by the cytoplasm of SMP neurons of the human colon and by vascular endothelial cells and pericytes (Supplementary Figure 1).

### ACE2 Immunoreactivity in the Tiger

Although the tissues of the tiger showed a certain degree of hyperemia, ACE2-IR was observed in all the tracts analyzed. However, the ACE2 immunolabeling was weaker with respect to the cats; ACE2-IR was not observed in the smooth muscle cells of the *muscularis mucosae*, the *tunica muscularis*, and the blood vessels.

**Figure 3:**
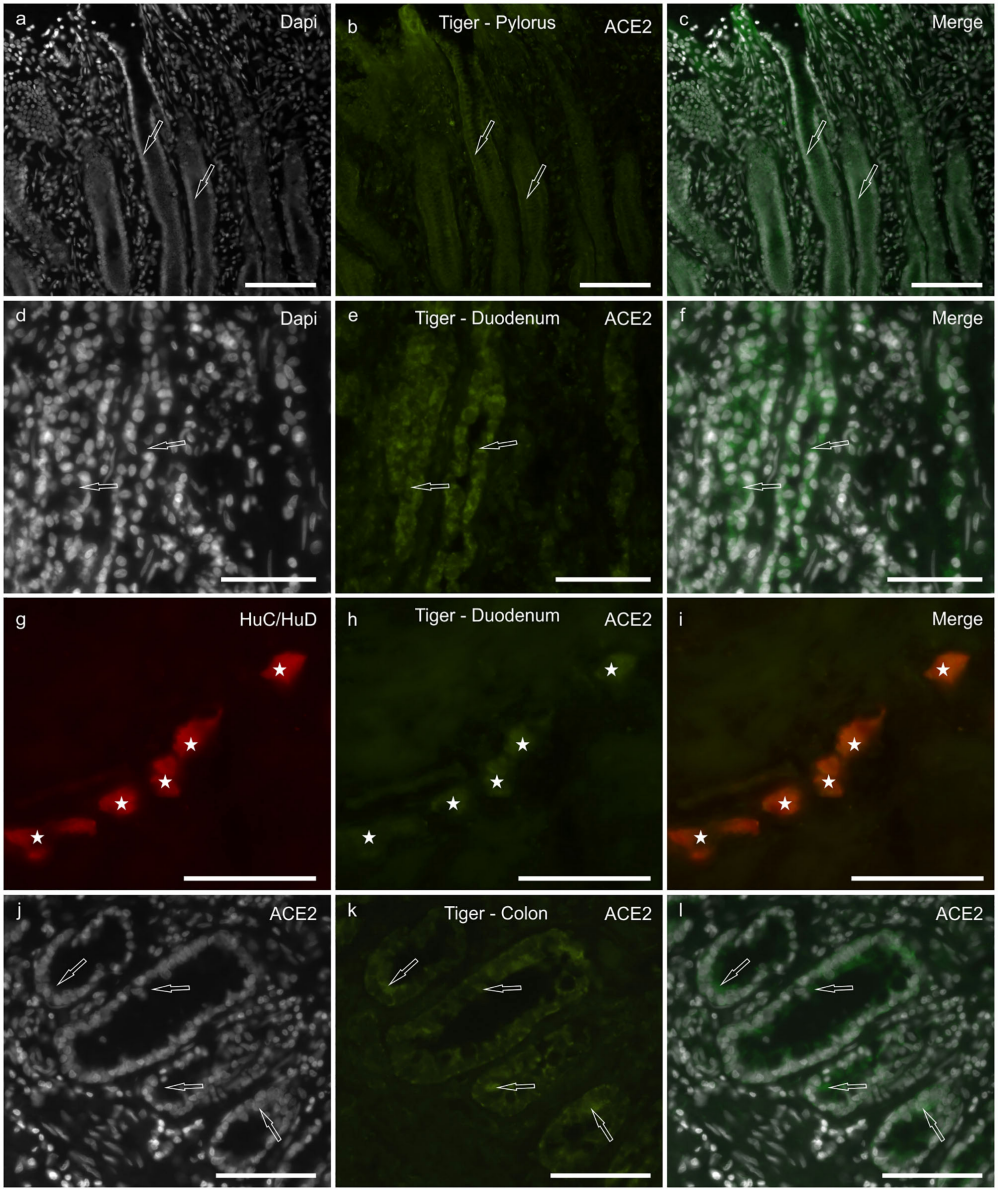
Photomicrographs of cryosections of the pylorus **(a–c)**, duodenum **(d–i)**, and colon **(j–l)** of the tiger showing angiotensin-converting enzyme 2 immunoreactivity (ACE2-IR). **(a–c)** Arrows indicate two pyloric glands in which weak ACE2-IR was expressed by the epithelial cells. **(d–f)** Arrows indicate two duodenal crypts in which epithelial cells expressed moderate ACE2-IR. **(g–i)** Stars indicate the submucosal plexus neurons of the duodenum co-expressing ACE2- and HuC/HuD-immunoreactivity. **(j–l)** Arrows indicate some epithelial cells of the colonic intestinal glands expressing moderate ACE2-IR. Bar: 100 μm.

In the stomach, faint ACE2-IR was expressed by the epithelial cells of the pyloric glands (Figures 3a–c) and the MP neurons (data not shown). In the duodenum, moderate ACE2-IR was observed in the epithelial cells of the crypts (Figures 3d–f). As observed in the cats, the MP and the SMP neurons (Figures 3g–i) showed cytoplasmic ACE2-IR. In the colon, moderate ACE2-IR was observed in the epithelial cells of the intestinal glands (Figures 3j–l).

## Discussion

To the best of the Authors' knowledge, the present study is the first demonstration of ACE2 localization in the feline (cat and tiger) GIT. A human-specific anti-ACE2 antibody was utilized, also considering that a recent biomolecular study demonstrated the homology of cat ACE2 with human ACE2 (12). The findings of the present research were partially consistent with those obtained in the human GIT in which ACE2-IR has been observed in the cytoplasm of the epithelial cells, and the smooth cells of the blood vessels, the *muscularis mucosae* and the *tunica muscularis* (5, 7). In the current study ACE2-IR was undetectable in the GIT goblet cells; this evidence is in contrast to what observed in the cat respiratory tract, in which the tracheo-bronchial goblet cells strongly expressed ACE2-IR (18) or in the human upper airway, in which nasal goblet cells express ACE2 gene, together with a number of genes associated with immune functions (19).

The localization of ACE2-IR in the gastrointestinal mucosa of the cats and the tiger anatomically suggested that SARS-CoV-2 can bind to its unique receptor on host cells; however, this finding alone did not necessarily mean that SARS-CoV-2 could replicate in the feline GIT. Once again, the positivity of the cat and the tiger fecal samples to SARS-CoV-2 (13, 14) confirmed that the feline GIT could be an important replication site of the virus which could consequently spread the infection by means of the stool.

Since SARS-CoV is completely inactivated by gastric pH (20), it is plausible that the replication of SARS-CoV-2 in the GIT may have become resistent to an increase in the gastric pH due to atrophic gastritis, gastric intestinal metaplasia, *Helicobacter pylori* infection, or the prolonged use of proton pump inhibitors (5, 21). The presence of the coronavirus in the feces cannot be justified only by the swallowing saliva containing the virus because fecal swabs often remain positive even when pharyngeal swabs are negative (21, 22).

The evidence of ACE2-IR in the feline and human enteric neurons is a intriguing novelty, although not completely unexpected, since ACE2 has already been localized in the cytoplasm of the brain neurons of transgenic mice (23), and that the involvement of the central and peripheral nervous systems during Covid-19 are increasingly being recognized (24). However, it is known that one of the first symptoms of Covid-19 is the olfactory dysfunction, due to the replication of the virus in the olfactory mucosa, and that researchers have emphasized the need to investigate SARS-CoV-2 spread in the nervous system (25). Diarrhea, which was one of the most common findings during the course of the SARS-CoV illness (26), seems to also be an hallmark of Covid-19 (27). In tissue specimens of the GITs of SARS-CoV patients, microscopic examination has not detected any evident pathological changes in the mucosa (5, 26). The expression of ACE2-IR in the enteric neurons could suggest that diarrhea in SARS-CoV-2 humans and cats could be neurogenic since it is known that the enteric nervous system, in particular the neurons of the submucosal plexus, can regulate the majority of physiological gastrointestinal functions, including water secretion (28, 29).

The present study described the expression of the ACE2 receptor in the stomach and intestine of the cat and tiger for the first time and highlighted the GIT as a potential site of SARS-CoV-2 replication. The findings in the present study could serve as an anatomical basis for additional studies which consider the risk of fecal-oral transmission of the SARS-CoV-2 between cats, and between cats and humans. The expression of the ACE2 receptor in the enteric neurons could support the potential neurotropic properties of SARS-CoV-2.

## Data Availability Statement

All datasets generated for this study are included in the article/Supplementary Material.

## Ethics Statement

Ethical review and approval was not required for the animal study because According to the Directive 2010/63/EU of the European Parliament and of the Council of 22 September 2010 on the protection of animals used for scientific purposes, the Italian legislation (D. Lgs. n. 26/2014) does not require any approval by competent authorities or ethics committees, because this research did not influence any therapeutic decisions. Written informed consent was obtained from the owners for the participation of their animals in this study.

## Author Contributions

Study concept and design: RC, MP, and FF. The immunohistochemical experiments were carried out by: GG and FG. Acquisition of data: RC and GG. Drafting of the manuscript and study supervision: RC. All authors interpreted the data. All authors contributed to the revision of the article for critical intellectual content and have approved the final version.

## Conflict of Interest

The authors declare that the research was conducted in the absence of any commercial or financial relationships that could be construed as a potential conflict of interest.

## References

[1] Hashimoto T Perlot T Rehman A Trichereau J Ishiguro H Paolino M. ACE2 links amino acid malnutrition to microbial ecology and intestinal inflammation. Nature. (2012) 487:477–81. 10.1038/nature11228 22837003 PMC7095315

[2] Hoffmann M Kleine-Weber H Schroeder S Krüger N Herrler T Erichsen S The novel coronavirus 2019 (2019-nCoV) uses the SARS-coronavirus receptor ACE2 and the cellular protease TMPRSS2 for entry into target cells. bioRxiv. (2020) 181:271–80. 10.1101/2020.01.31.929042

[3] Huang C Wang Y Li X Ren L Zhao J Hu Y. Clinical features of patients infected with 2019 novel coronavirus in Wuhan, China. Lancet. (2020) 395:497–506. 10.1016/S0140-6736(20)30183-5 31986264 PMC7159299

[4] Zhou P Yang XL Wang XG Hu B Zhang L Zhang W. A pneumonia outbreak associated with a new coronavirus of probable bat origin. Nature. (2020) 579:270–3. 10.1038/s41586-020-2012-7 32015507 PMC7095418

[5] Xiao F Tang M Zheng X Liu Y Li X Shan H. Evidence for gastrointestinal infection of SARS-CoV-2. Gastroenterology. (2020) 158:1831–3.e3. 10.1053/j.gastro.2020.02.055 32142773 PMC7130181

[6] Chan PK To KF Lo AW Cheung JL Chu I Au FW. Persistent infection of SARS coronavirus in colonic cells *in vitro*. J Med Virol. (2004) 74:1–7. 10.1002/jmv.20138 15258961 PMC7166317

[7] Hamming I Timens W Bulthuis ML Lely AT Navis GJ van Goor H. Tissue distribution of ACE2 protein, the functional receptor for SARS coronavirus. A first step in understanding SARS pathogenesis. J Pathol. (2004) 203:631–7. 10.1002/path.1570 15141377 PMC7167720

[8] Gu J Han B Wang J. COVID-19: gastrointestinal manifestations and potential fecal-oral transmission. Gastroenterology. (2020) 158:1518–9. 10.1053/j.gastro.2020.02.054 32142785 PMC7130192

[9] Decaro N Lorusso A. Novel human coronavirus (SARS-CoV-2): A lesson from animal coronaviruses. Vet Microbiol. (2020) 244:108693. 10.1016/j.vetmic.2020.108693 32402329 PMC7195271

[10] Luan J Jin X Lu Y Zhang L. SARS-CoV-2 spike protein favors ACE2 from bovidae and cricetidae. J Med Virol. (2020). [Epub ahead of print]. 10.1002/jmv.25817. 32239522 PMC7228376

[11] Shi J Wen Z Zhong G Yang H Wang C Huang B. Susceptibility of ferrets, cats, dogs, and other domesticated animals to SARS-coronavirus 2. Science. (2020) 368:1016–20. 10.1126/science.abb7015 32269068 PMC7164390

[12] Qiu Y Zhao YB Wang Q Li JY Zhou ZJ Liao CH. Predicting the angiotensin converting enzyme 2 (ACE2) utilizing capability as the receptor of SARS-CoV-2. Microbes Infect. (2020) 22:221–5. 10.20944/preprints202003.0091.v1 32199943 PMC7156207

[13] Almendros A Gascoigne E Can companion animals become infected with Covid-19? Vet Rec. (2020) 86:419–420. 10.1136/vr.m1322 32245870

[14] Halfmann PJ Hatta M Chiba S Maemura T Fan S Takeda M. Transmission of SARS-CoV-2 in domestic cats. N Engl J Med. (2020) 383:592–4. 10.1056/NEJMc2013400 32402157 PMC9678187

[15] Chini M Coronavirus: Belgian Cat Infected by Owner. The Brussels Times (2020). Available online at: www.brusselstimes.com/all-news/belgium-allnews/103003/coronavirus-belgian-woman-infected-her-cat

[16] Li X. Can cats become infected with Covid-19? Vet Rec. (2020) 186:457–8. 10.1136/vr.m1455 32299985

[17] Giancola F Torresan F Repossi R Bianco F Latorre R Ioannou A. Downregulation of neuronal vasoactive intestinal polypeptide in Parkinson's disease and chronic constipation. Neurogastroenterol Motil. (2017) 29:e12995. 10.1111/nmo.12995 27891695 PMC5393951

[18] Van den Brand JM Haagmans BL Leijten L van Riel D Martina BE Osterhaus AD. Pathology of experimental SARS coronavirus infection in cats and ferrets. Vet Pathol. (2008) 45:551–62. 10.1354/vp.45-4-551 18587105

[19] Sungnak W Huang N Bécavin C Berg M Queen R Litvinukova M. SARS-CoV-2 entry factors are highly expressed in nasal epithelial cells together with innate immune genes. Nat Med. (2020) 26:681–7. 10.1038/s41591-020-0868-6 32327758 PMC8637938

[20] Darnell ME Subbarao K Feinstone SM Taylor DR. Inactivation of the coronavirus that induces severe acute respiratory syndrome, SARS-CoV. J Virol Methods. (2004) 121:85–91. 10.1016/j.jviromet.2004.06.006 15350737 PMC7112912

[21] Uno Y. Why does SARS-CoV-2 invade the gastrointestinal epithelium? Gastroenterology. (2020). [Epub ahead of print]. 10.1053/j.gastro.2020.04.006. 32283099 PMC7194682

[22] Smyk W Janik MK Portincasa P Milkiewicz P Lammert F Krawczyk M. COVID-19: focus on the lungs but do not forget the gastrointestinal tract. Eur J Clin Invest. (2020) e13276. 10.1111/eci.13276. [Epub ahead of print]. 32406522 PMC7261996

[23] Doobay MF Talman LS Obr TD Tian X Davisson RL Lazartigues E. Differential expression of neuronal ACE2 in transgenic mice with overexpression of the brain renin-angiotensin system. Am J Physiol Regul Integr Comp Physiol. (2007) 292:R373–81. 10.1152/ajpregu.00292.2006 16946085 PMC1761128

[24] Natoli S Oliveira V Calabresi P Maia LF Pisani A. Does SARS-Cov-2 invade the brain? Translational lessons from animal models. Eur J Neurol. (2020). [Epub ahead of print]. 10.1111/ene.14277. 32333487 PMC7267377

[25] Butowt R Bilinska K. SARS-CoV-2: olfaction, brain infection, and the urgent need for clinical samples allowing earlier virus detection. ACS Chem Neurosci. (2020) 11:1200–3. 10.1021/acschemneuro.0c00172 32283006

[26] Gu J Korteweg C Pathology and pathogenesis of severe acute respiratory syndrome. Am J Pathol. (2007) 170:1136–47. 10.2353/ajpath.2007.061088 17392154 PMC1829448

[27] Agarwal A Chen A Ravindran N To C Thuluvath PJ. Gastrointestinal and liver manifestations of COVID-19. J Clin Exp Hepatol. (2020) 10:263–5. 10.1016/j.jceh.2020.03.001 32405183 PMC7212283

[28] Furness JB The Enteric Nervous System. Oxford: Blackwell (2006). 129 p.

[29] Kleinschmidt S Nolte I Hewicker-Trautwein M. Structural and functional changes of neuronal and glial components of the feline enteric nervous system in cats with chronic inflammatory and non-inflammatory diseases of the gastrointestinal tract. Res Vet Sci. (2011) 91:e129–35. 10.1016/j.rvsc.2011.01.026 21349562

