# Supplementary material for: ACE2 Expression in the Cat and the Tiger Gastrointestinal Tracts
Source: Front Vet Sci. 2020 Aug 13;7:514. doi: 10.3389/fvets.2020.00514 (PMC7438561; doi:10.3389/fvets.2020.00514)
Supplement: Supplementary file 1 [file Image_1.pdf]

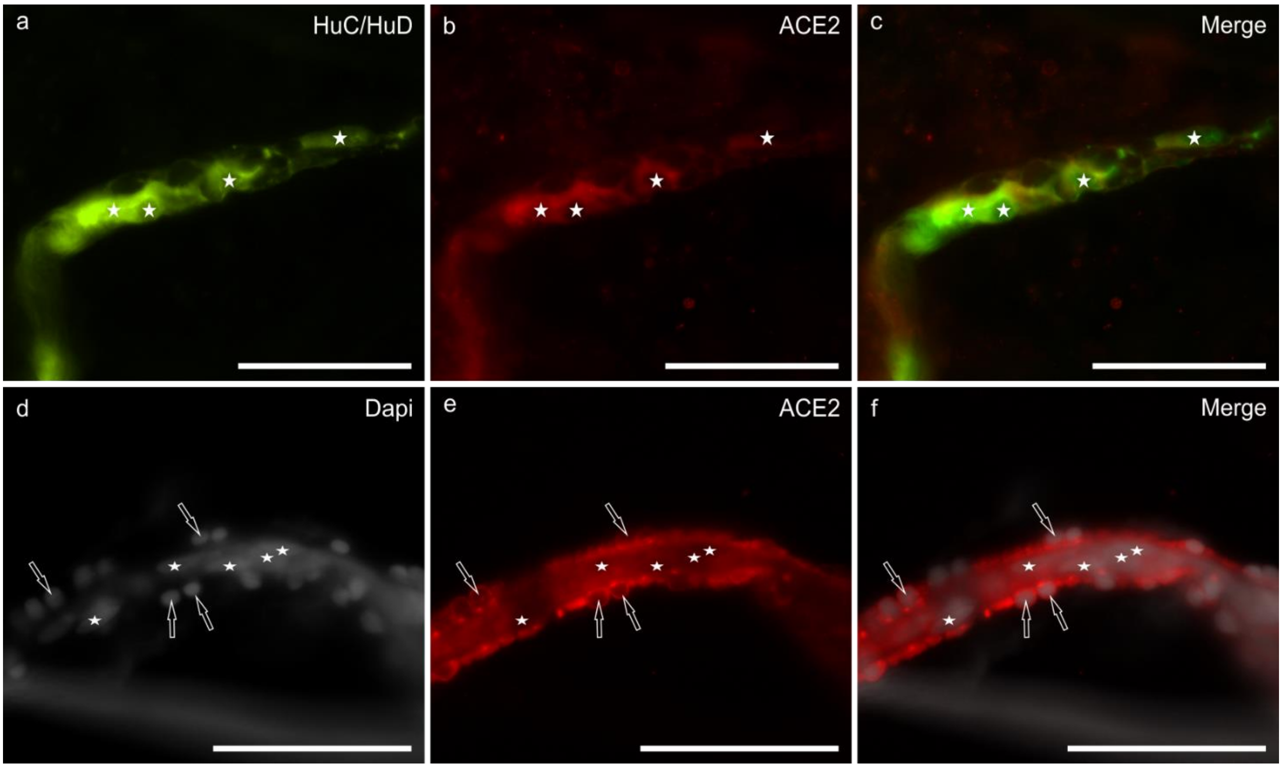

Supplementary Fig. 1. a-f) Photomicrographs showing immunoreactivity for angiotensin-converting enzyme 2 (ACE2) in submucosal wholemount preparation of human colon. a-c) Stars indicate submucosal plexus HuC/HuD immunoreactive neurons (a) co-expressing bright ACE2 immunoreactivity (b). d-f) Stars and arrows indicate the nuclei of vascular endothelial cells and pericytes, respectively, which expressed bright ACE2 immunoreactivity.

Bar: a-f= 100 µm
